# Target of Rapamycin (TOR) Negatively Regulates Ethylene Signals in *Arabidopsis*

**DOI:** 10.3390/ijms21082680

**Published:** 2020-04-12

**Authors:** Fengping Zhuo, Fangjie Xiong, Kexuan Deng, Zhengguo Li, Maozhi Ren

**Affiliations:** 1 School of Life Sciences, Chongqing University, Chongqing 401331, China; zfp19730129@126.com (F.Z.); xiongfj2009@126.com (F.X.); wmjbrj@cqu.edu.cn (K.D.); 2 School of Chemistry and Chemical Engineering, Chongqing University of Science and Technology, Chongqing 401331, China; 3 Zhengzhou Research Base, State Key Laboratory of Cotton Biology, Zhengzhou University, Zhengzhou 455001, China; 4 Institute of Urban Agriculture, Chinese Academy of Agricultural Sciences, Chengdu 610000, China

**Keywords:** target of rapamycin, gene expression, hypocotyl growth inhibition, TAP46-ACS2/6 interaction, ethylene signals, *Arabidopsis*

## Abstract

Target of rapamycin (TOR) acts as a master regulator in coordination of cell growth with energy and nutrient availability. Despite the increased appreciation of the essential role of the TOR complex in interaction with phytohormone signaling, little is known about its function on ethylene signaling. Here, through expression analysis, genetic and biochemical approaches, we reveal that TOR functions in the regulation of ethylene signals. Transcriptional analysis indicates that TOR inhibition by AZD8055 upregulated senescence- and ethylene-related genes expression. Furthermore, ethylene insensitive mutants like *etr1-1*, *ein2-5* and *ein3 eil1*, showed more hyposensitivity to AZD8055 than that of WT in hypocotyl growth inhibition. Similarly, blocking ethylene signals by ethylene action inhibitor Ag+ or biosynthesis inhibitor aminoethoxyvinylglycine (AVG) largely rescued hypocotyl growth even in presence of AZD8055. In addition, we also demonstrated that Type 2A phosphatase-associated protein of 46 kDa (TAP46), a downstream component of TOR signaling, physically interacts with 1-aminocy-clopropane-1-carboxylate (ACC) synthase ACS2 and ACS6. *Arabidopsis* overexpressing ACS2 or ACS6 showed more hypersensitivity to AZD8055 than WT in hypocotyl growth inhibition. Moreover, ACS2/ACS6 protein was accumulated under TOR suppression, implying TOR modulates ACC synthase protein levels. Taken together, our results indicate that TOR participates in negatively modulating ethylene signals and the molecular mechanism is likely involved in the regulation of ethylene biosynthesis by affecting ACSs in transcription and protein levels.

## 1. Introduction

Target of rapamycin (TOR), an evolutionarily conserved serine/threonine protein kinase, plays an essential role in controlling cell growth and maintaining cellular homeostasis in response to nutrition and energy availability in eukaryotes [1,2,3]. The elements of the TOR signaling pathway are also highly conserved in eukaryote organisms from yeast to humans, but the chief players differ comparatively between heterotrophic species and photoautotrophic plants [4,5,6,7]. To date, at least two structurally and functionally distinct TOR protein complexes have been characterized in eukaryotes [2,8,9,10]. In mammals, TOR protein forms two different TOR complexes (TORC1 and TORC2) by interacting with two regulatory partners. TORC1 and TORC2 share a common subunit, small lethal with SEC13 protein 8 (LST8), while differ from two representative regulatory subunits, regulatory-associated protein of mTOR (RAPTOR) and rapamycin-insensitive companion of mTOR (RICTOR), which participate in formation of TORC1 and TORC2, respectively [2,4]. In plants, TOR, LST8 and RAPTOR orthologues was found in all sequenced plant species, while RICTOR orthologue is yet to be identified in plant genomes, therefore whether plants have TORC2 remains to be verified [1,5,7,11]. Previous works show that some canonical downstream components of TOR signaling are conserved in plants, such as the ribosomal protein S6 kinase (S6K), which was reported to activate protein synthesis by phosphorylating ribosomal S6 proteins (RPS6), and the Type 2A phosphatase-associated protein of 46 kDa (TAP46), which regulates cell growth in coordination with nutrient and environmental conditions as well as the repression of programmed cell death or senescence [12,13,14].

The lethality of TOR function disruption in eukaryotic organisms has prevented dissection of the TOR functions, especially in plant species [15,16,17]. Rapamycin (an allosteric inhibitor of TOR) and active-siteTOR inhibitors (asTORis) have been used to dissect the TOR signaling pathway in yeast, animals and plants [18,19,20,21,22]. Although plants respond poorly to rapamycin mainly because plant FK506-binding protein 12 kD (FKBP12) has lost key amino acids to the interaction with rapamycin, over-expression of yeast or human FKBP12 can restores rapamycin sensitivity in *Arabidopsis* [23,24,25,26,27,28]. Importantly, this rapamycin-FKBP12 system confers a highly inducible, selective and reversible system to explore TOR functions in plants [24,28,29]. Unlike rapamycin, asTORis is a kind of ATP competitive TOR inhibitors which can target the ATP-binding pocket of the kinase domain of both mTORC1 and mTORC2 complexes, and thus have broader inhibitory effects of mTOR than rapamycin [19,20]. Thus, asTORis like the AZD8055, Torin1, KU63794 are extensively used to illuminate the TOR signaling pathway in plants [20,21,27,29,30].

In recent years, studies involved in crosstalk between TOR and phytohormone signaling are extensively described [31,32]. Previously, Dong et al. reported that genes related to the main phytohormones signaling were differently regulated under TOR inhibition in *Arabidopsis*. They also note that plant hormones that promote growth, such auxin, brassinosteroids (BR), cytokinins (CK) and gibberellins (GA) are positively correlated with TOR signaling, while abscisic acid (ABA), ethylene, jasmonates(JA) and salicylic acid (SA) signal pathways have the opposite relationship with TOR [33]. TOR was reported to integrate auxin and nutrient signaling to regulate translation reinitiation of the mRNAs that contain upstream open reading frames (uORFs), such as auxin response factors [14]. Through the small GTPase ROP2 (Rho-like GTPases), auxin, also as a direct upstream effector, activates TOR signaling [34]. Later study shows that TOR also participates in regulating brassinosteroid signaling through a downstream effector S6K2 interacts and phosphorylates BRASSINOSTERIOD INSENSITIVE 2 (BIN2), the negative regulator in BR signaling [29]. Additionally, autophagy also has been reported to link TOR and BRASSINAZOLE-RESISTANT 1 (BZR1) in response to carbon availability [35]. A recent study addressed a crosstalk of reciprocal regulation between TOR and ABA signaling in balancing plant growth and stress [36]. Moreover, TOR negatively regulates JA biosynthesis both in *Arabidopsis* and cotton [37]. Regarding the hormones ethylene, our previous date show that the genes involved in the ethylene signaling pathway were all up-regulated under TOR inactivated by AZD8055, implying possible interaction between TOR and ethylene signaling exists in plants, however, little is known about the involved molecular mechanisms.

Ethylene is a gaseous plant hormone and plays an essential role in regulation of plant growth, development, senescence and stresses responses [38,39,40,41]. Although there exist distinct signal transduction pathways of ethylene especially in response to nutrient deficiencies [41,42,43,44,45], a largely linear ethylene signaling pathway has established in plants [46,47]. Ethylene is perceived by a five-member family endoplasmic reticulum (ER)-localized receptors [48,49]. Once ethylene gas binds to the receptors, both receptors and CONSTITUTIVE RESPONSE1 (CTR1) are inactivated. CTR1 is a Raf-like Ser/Thr protein kinase that acts as a negative regulator of ethylene signaling [50]. Upon CTR1 inactivation, ETHYLENE INSENSITIVE 2 (EIN2) cannot be phosphorylated and subsequently is subjected to site-specific proteolysis, resulting in the C-terminal EIN2 which is translocated to the nucleus and activate the key transcription factor ETHYLENE-INSENSITIVE 3 (EIN3) and EIN3-Like 1 (EIL1) [50,51,52,53,54,55]. Ethylene biosynthesis starts from S-adenosylmethionine (SAM), which is converted to 1-aminocy-clopropane-1-carboxylate (ACC) by the enzyme ACC synthase (ACS), which is generally considered as a rate-limiting step. Then ACC is oxidized by the ACC oxidase (ACO) to produce ethylene [56,57,58]. The *Arabidopsis* genome contains 12 annotated *ACS* genes, eight of which (*ACS2*, *ACS4-9* and *ACS11*) encode ACC synthase with functional activities, and are divided into three types based on their conserved and variant C-terminus domain [56,57,58]. Generally, the expression of the *ACS* genes is regulated by various signals and active ACC synthase is labile and present at low levels to tightly controlled ethylene biosynthesis. Therefore, ethylene production in plants is maintained at a low basal level, but it is induced rapidly and significantly at some stages of development or in response to environmental stimuli [57,58].

In this study, we reanalyzed our previous transcriptome data and found that TOR inhibition upregulated senescence-related gene expression, accompanying with upregulating expression of ethylene biosynthetic and response genes. The results imply the potential crosstalk between TOR and ethylene signaling. Through genetic and biochemical approaches, we found TOR functions in the regulation of ethylene biosynthesis. In addition, we also demonstrated that TAP46, a downstream component of TOR, physically interacts with ACS2/ACS6, and the ACS2/ACS6 protein are accumulated under TOR inhibition. The results of this study establish a link between TOR and ethylene signaling, in which TOR negatively modulates ethylene signals and the molecular mechanism is likely involved in ethylene biosynthesis by regulating ACSs in transcription and protein levels.

## 2. Results

### 2.1. TOR Inhibition Upregulates Senescence- and Ethylene-Related Genes Expression

**Figure 1:**
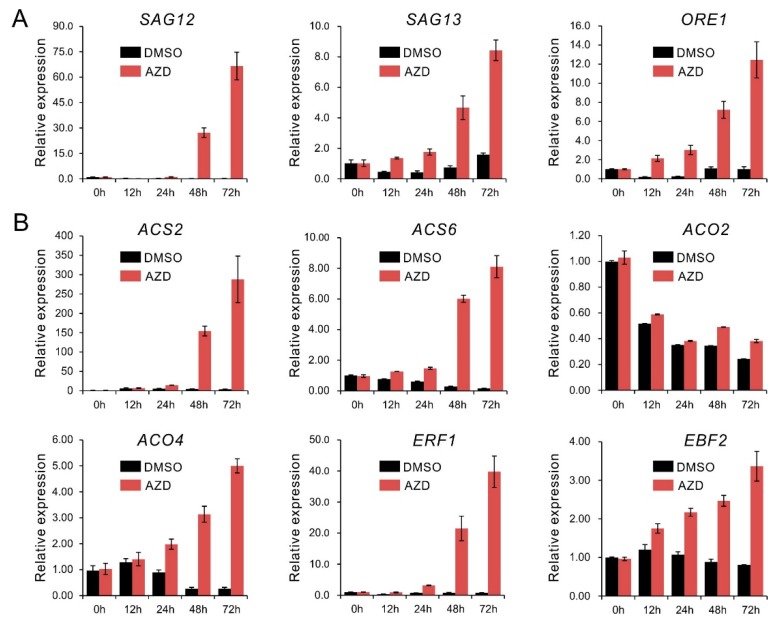
qRT-PCR analyses of the senescence- and ethylene-related genes expression under target of rapamycin (TOR) inhibition. (**A**) Senescence marker genes, (**B**) the representative genes involved in ethylene biosynthesis and response. Ten-day-old seedlings were transferred to liquid 1/2MS medium supplemented with 2 μM AZD8055 (AZD) or DMSO (solvent control) for incubation as the indicated time. Expression levels were normalized to an internal control *ACTIN2*. Data are represented as means ± SD (*n* = 3).

Although it has been reported that rapamycin can significantly delay senescence in *FKBP12*-expressing plants [28], many studies showed that severe suppression of TOR accelerated leaves chlorosis or senescence in the growing plants [13,33,59]. To understand how TOR affects plant senescence, here we reanalyzed our previous RNA sequencing data from *Arabidopsis* seedlings treated by 2 μM AZD8055 (DMSO as the solvent control). The results showed that there were abundant senescence associated genes including the marker gene *SAG12* and *SAG13*, displayed consistently up-regulated expression under TOR inhibition, implying TOR negatively regulates senescence genes expression to some extent (Table S1). Furtherly, expression analysis by qPCR also showed consistent results, the representative genes of senescence such as *SAG12*, *SAG13* and *ORE1* exhibited an increasing tendency in transcript level with time-scale extending in presence of 2 μM AZD8055 (Figure 1A). Based on the transcriptome data, our previous work showed that TOR plays a key role in regulating hormones signaling in which ethylene signaling also was exhibited [33]. Considering the function of ethylene in regulating plant senescence, we speculated that ethylene signaling was associated with the senescence caused by TOR inhibition. Further scanning this data, we found that many ethylene associated genes involved in biosynthesis and response displayed consistent up-regulation under TOR inhibition. Expression analysis by qPCR further validated these results that the representative genes of ethylene biosynthesis (*ACS2*, *ACS6*, *ACO2* and *ACO4*) and response (*ERF1* and *EBF2*) were obviously upregulated by 2 μM AZD8055 with time-scale extending (Table S1, Figure 1B). In addition, *ORE1/NAC2*, *NAP* and *SGR1* (STAY-GREEN1, also known as *NYE1*), which are usually taken as marker genes to monitor ethylene-mediated senescence [60,61,62,63], also displayed up-regulated expression under TOR inhibition (Table S1). These results imply that TOR is involved in the regulation of ethylene signaling.

### 2.2. Ethylene Insensitive Mutants Display Hyposensitivity to AZD8055

**Figure 2:**
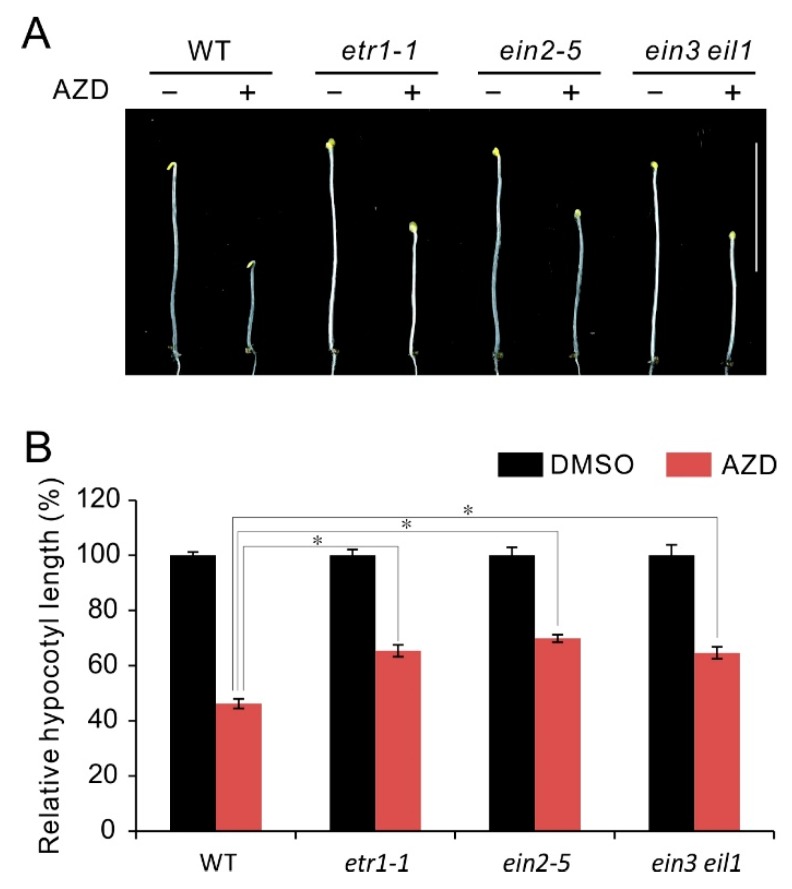
Phenotype of ethylene insensitive mutants in response to TOR inhibitor AZD8055. (**A**) Phenotype of 5-day-old etiolated seedlings of WT, *etr1-1*, *ein2-5* and *ein3 eil1* double mutant grown in the presence of 1 μM AZD8055 under dark condition. (**B**) Quantitative analyses of relative hypocotyl length (AZD8055 vs. DMSO). * *p* < 0.05 (Student’s *t* test). Error bars indicate the SEM (*n* ≥ 40). Bar = 1 cm.

Seedling triple response with shortening of hypocotyl is an important characteristic of ethylene response [64]. In etiolated *Arabidopsis* seedlings, ethylene or its precursor ACC prevents hypocotyls elongation, while have no effect on hypocotyl growth of ethylene insensitive mutants [46,64]. Previous studies show that TOR also plays an essential role in hypocotyl growth, and TOR inhibitors can effectively repressed hypocotyl growth in etiolated seedlings [28,35]. In order to further explore the regulatory relationship between TOR and ethylene signaling, here we checked the response of hypocotyl growth in ethylene insensitive mutants *etr1-1*, *ein2-5* and *ein3 eil1* to TOR inhibitor AZD8055. As shown in Figure 2A, all the checked seedlings showed hypocotyl growth inhibition under AZD8055 treatment in comparison to control treatment, while these mutants *etr1-1*, *ein2-5* and *ein3 eil1* displayed weaker sensitivity than WT to AZD8055 in hypocotyl growth inhibition. Correspondingly, the calculations of relative hypocotyl length (AZD8055/DMSO) in *etr1-1*, *ein2-5* and *ein3 eil1* is about 65%, 69% and 64%, respectively, which is remarkably higher than that in WT (about 46%) (Figure 2B). The results suggest hypocotyl growth inhibition caused by TOR suppression is partly dependent on ethylene signaling and imply TOR negatively regulates ethylene signals.

### 2.3. Activated Ethylene Signal Is Required for Hypocotyl Growth Inhibition of TOR Inactivity

**Figure 3:**
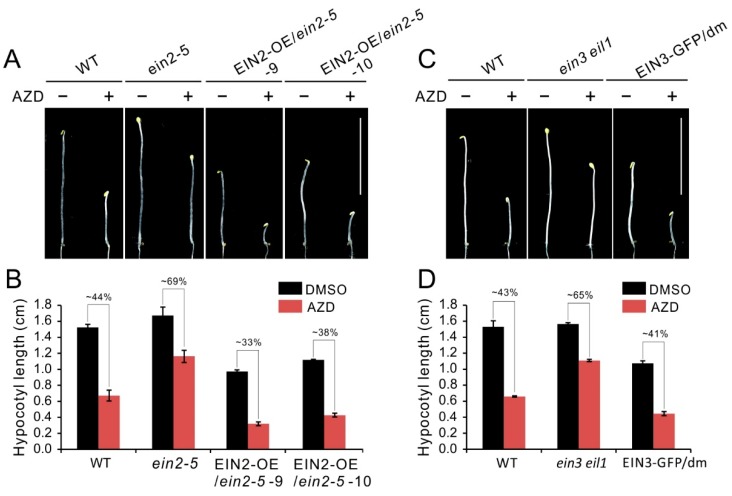
Hypocotyl growth inhibition caused by TOR repression partly depends on intact ethylene signaling. (**A**) Phenotype of 5-day-old etiolated seedlings of WT, *ein2-5* and EIN2 overexpressing plants on the *ein2-5* background grown in the presence of 1 μM AZD8055 under dark condition. (**B**) Quantitative analyses of hypocotyl length. The numbers represent the percentage of hypocotyl length in treatment of AZD8055 compared to DMSO. Error bars indicate the SEM (*n* ≥ 40). (**C**) Phenotype of 5-day-old etiolated seedlings of WT, *ein3 eil1* and EIN3 overexpressing plants on the *ein3 eil1* double mutant (dm) background grown in the presence of 1 μM AZD8055 under dark condition. (**D**) Quantitative analyses of hypocotyl length. The numbers represent the percentage of hypocotyl length in treatment of AZD8055 compared to DMSO. Error bars indicate the SEM (*n* ≥ 40). Bar = 1 cm.

To investigate hypocotyl growth inhibition of TOR inactivity whether depends on intact ethylene signaling, we generated EIN2 overexpressing plants on the *ein2-5* background. Compared with the *ein2-5* or WT etiolated seedlings, the EIN2 overexpressing lines (EIN2-OE/*ein2-5*) had obvious shorter hypocotyl under control condition, suggesting overexpression of EIN2 recovered and enhanced activation of ethylene in *ein2-5* mutant (Figure 3A,B). Furthermore, EIN2-OE/*ein2-5* lines showed more sensitivity to AZD8055 and had more severely reduction in hypocotyl length compared that of *ein2-5* or WT plants (Figure 3A). Concretely, upon AZD8055 treatment, the relative hypocotyl length of WT was about 44%, and this parameter is significantly lower than that of *ein2-5* (about 69%). However, overexpression of EIN2 in *ein2-5* plants caused this parameter dramatically reduction to 33% and 38% in transgenic line 9 and 10, respectively (Figure 3B). Similar results also were observed in EIN3-GFP/dm plants, as shown in Figure 3C,D, overexpression of EIN3 in *ein3 eil1* mutant enhanced sensitivity of *ein3 eil1* to AZD8055. Taken together, the above results suggest that hypocotyl growth inhibition of TOR inactivity is partly dependent on intact ethylene signaling.

### 2.4. Ethylene Inhibitors Relieve Hypocotyl Growth Inhibition under TOR Inactivity

**Figure 4:**
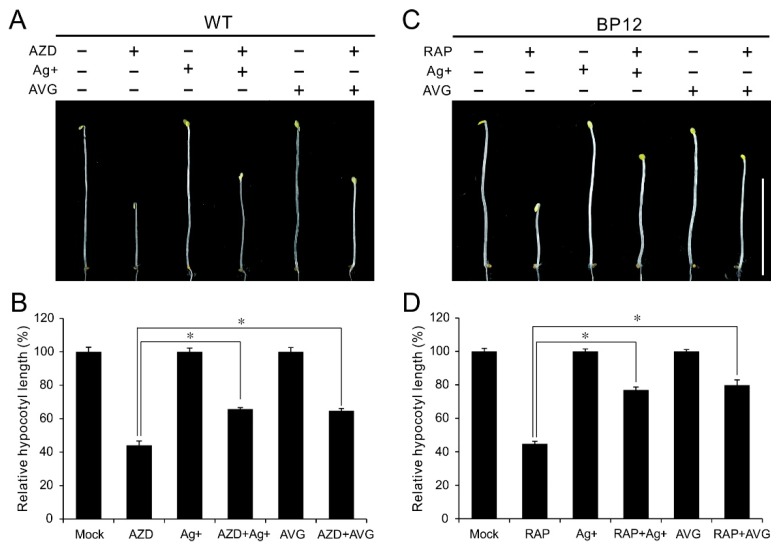
Ethylene inhibitor antagonizes TOR inhibitors in the regulation of hypocotyl growth. (**A**) Phenotype of 5-day-oldetiolated WT seedlings grown in 1/2 MS medium with 1 μM AZD8055, 10 μM Ag+, 5 μM AVG or their combination under dark condition. (**B**) Quantitative analyses of relative hypocotyl length of various treatments compared to mock treatment. * *p* < 0.05 (Student’s *t* test). Error bars indicate the SEM (*n* ≥ 40). (**C**) Phenotype of 5-day-old etiolated BP12 (a *ScFKBP12*-expressing line) seedlings grown in 1/2 MS medium with 1 μM Rapamycin (RAP), 10 μM Ag+, 5 μM AVG or their combination. (**D**) Quantitative analyses of relative hypocotyl length of various treatments compared to mock treatment. * *p* < 0.05 (Student’s *t* test). Error bars indicate the SEM (*n* ≥ 40). Bar = 1 cm.

Ethylene inhibitors like the silver (Ag+), a potent inhibitor of ethylene action and aminoethoxyvinylglycine (AVG), an inhibitor of ACC synthase, have been extensively used to block ethylene signals. To further explore the mechanism underlying TOR in regulation of ethylene signaling, the ethylene inhibitor Ag+ and AVG were employed to treat WT or BP12 (Rapamycin hypersensitive transgenic line with *ScFKBP12*) plants under TOR inhibition. As shown in Figure 4A,B, both Ag+ and AVG had little effect on hypocotyl growth in etiolated WT seedlings, while these two ethylene inhibitor remarkably rescued the hypocotyl growth even in presence of 1 μM AZD8055. The relative hypocotyl length in etiolated WT seedlings is 44% in the presence of AZD8055, while it was increased to 65% and 64% by Ag+ and AVG, respectively (Figure 4B). Consistently, hypocotyl growth inhibition of TOR inactivity caused by rapamycin in etiolated BP12 seedlings also were notably restored by additional Ag+ or AVG (Figure 4C,D). The relative hypocotyl length in etiolated BP12 seedlings was recovered to 76% and 79% by Ag+ and AVG, respectively, even in the presence of rapamycin (Figure 4D). These results further confirmed that hypocotyl growth inhibition of TOR inactivity is dependent on activated ethylene signaling. Meanwhile, these results also imply that TOR regulates ACSs activity in protein level.

### 2.5. TAP46 Physically Interacts with ACS2/ACS6

**Figure 5:**
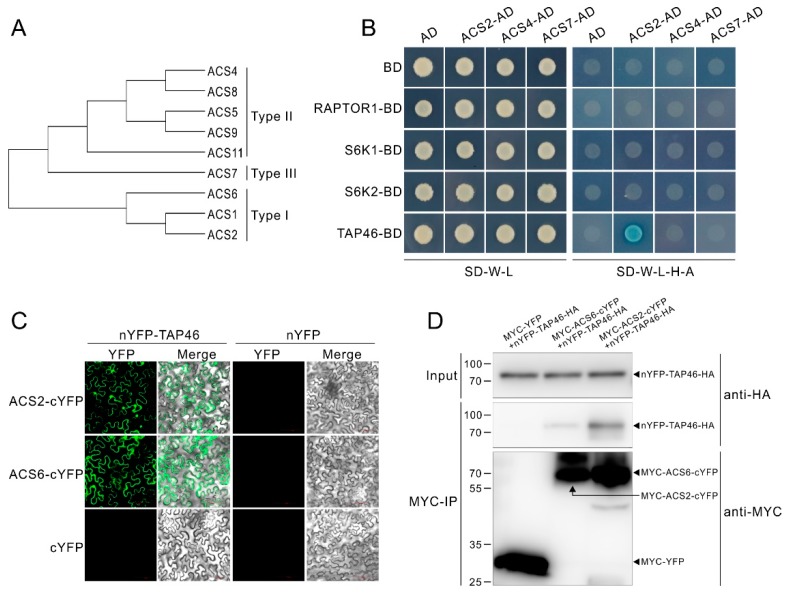
Type 2A phosphatase-associated protein of 46 kDa (TAP46) directly interacts with 1-aminocy-clopropane-1-carboxylate synthase (ACS)2 or ACS6. (**A**) Three type of ACSs. (**B**) Yeast two hybrid (Y2H) assays show putative interaction between TAP46 and ACS2. Yeast cells co-expressing the constructs were grown on basic (SD-W-L), and then selected selective media (SD-W-L-H-A) with Aureobasidin A and X-α-gal. (**C**) Bimolecular fluorescence complementation (BiFC) assays show TAP46 interacts with ACS2 and ACS6 in vivo. *Agrobacterium* carrying nYFP and cYFP constructs were mixed and transiently expressed in tobacco leaf epidermal cells (**D**) co-immunoprecipitation (Co-IP) assays confirm the physically interaction of TAP46 with ACS2 or ACS6. The constructs were co-transformed into tobacco leaf epidermal cells. Total proteins were immunoprecipitated with Anti-c-Myc Magnetic Beads and immunoblotted with anti-HA and anti-MYC antibodies.

Our above demonstration that AVG, as the inhibitor of ACSs, can antagonizes TOR inhibitors, suggests TOR activity affects ACC synthase activity in protein level. To further investigate the underlying molecular mechanism, we performed a small-scale interaction screen by yeast two hybrid assays (Y2H) between TOR signaling components and ACSs. In *Arabidopsis*, 8 ACSs (ACS2, ACS4-9 and ACS11) show functional activities, and these ACSs are divided into 3 types based on their C-terminal domain (Figure 5A). In addition, the ACS1, which was presumed to act as a regulator of ACS activity, is also classed in Type I [57]. For the Y2H assay, the ACS2, ACS4 and ACS7 were picked up as represents of the 3 type ACS, respectively. As shown in Figure 5B, just the TAP46 showed putative interaction with the ACS2. The result suggests that ACS6, belonging to the same type with the ACS2, is also a possible interacting factor of TAP46. We then performed an *in vivo* bimolecular fluorescence complementation (BiFC) assay by making constructs expressing TAP46-HA fused the amino (N)-terminal YFP (nYFP), and MYC-ACS2 or MYC-ACS6 fused the carboxy (C)-terminal YFP (cYFP). The results showed that co-expressing nYFP-TAP46-HA and MYC-ACS2/ACS6-cYFP, but not nYFP-TAP46-HA and MYC-YFP vector or nYFP-HA vector and MYC-ACS2/ACS6-cYFP, in tobacco leaves reconstituted punctuated YFP signals (Figure 5C). Next, we performed a co-immunoprecipitation (Co-IP) assay with the tobacco leaves to confirm the interaction of TAP46 with ACS2 or ACS6. The results showed that TAP46 was immunoprecipitated by ACS2 or ACS6, but not by the control protein YFP (Figure 5D), further indicating an interaction of TAP46 with ACS2 or ACS6. These results imply that TOR modulates ethylene biosynthesis through the molecular interaction between downstream effector of TOR and ACS2/ACS6 in protein level.

### 2.6. TOR Inhibition Causes Accumulation of ACS2/ACS6 Protein

**Figure 6:**
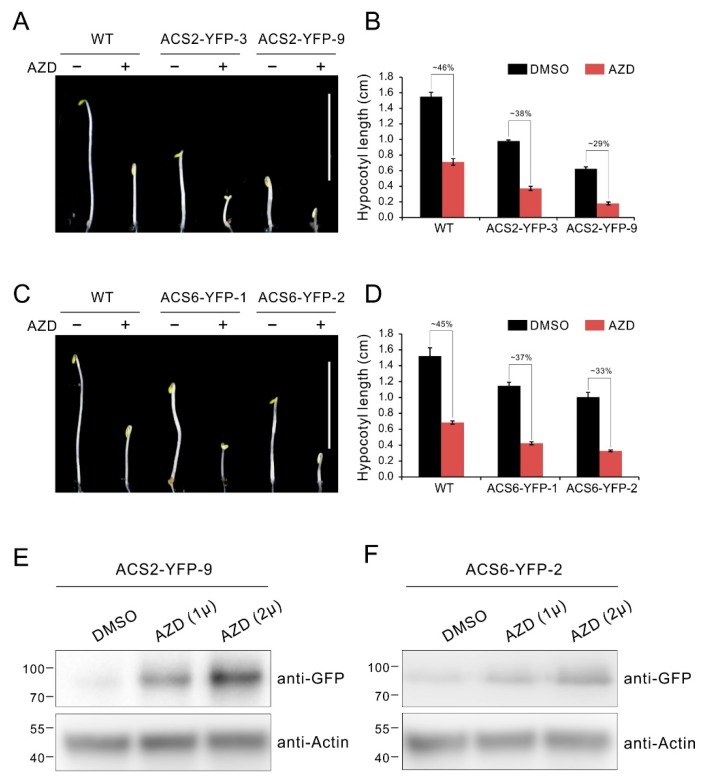
TOR inhibition causes accumulation of the ACS2/ACS6 protein. (**A**) Phenotype of 5-day-old etiolated seedlings of WT and ACS2 overexpressing lines grown in 1/2 MS medium supplied with 1 μM AZD8055 under dark condition. Bar = 1 cm. (**B**) Quantitative analyses of hypocotyl length. The numbers represent relative hypocotyl length in treatment of AZD8055 compared to DMSO. Error bars indicate the SEM (*n* ≥ 40). (**C**) Phenotype of 5-day-old etiolated seedlings of WT and ACS6 overexpressing lines grown in 1/2 MS medium supplied with 1 μM AZD8055 under dark condition. Bar = 1 cm. (**D**) Quantitative analyses of hypocotyl length. The numbers represent relative hypocotyl length in treatment of AZD8055 compared to DMSO. Error bars indicate the SEM (*n* ≥ 40). (**E**,**F**) Ten-day-old seedlings of ACS2 or ACS6 overexpressing lines were incubated in liquid 1/2 MS medium supplied with indicated dose of AZD8055 or DMSO (solvent control) for 24 h. ACS2 or ACS6 protein was immunoblotted with anti-GFP antibodies.

To further define a role of ACS2/ACS6 in TOR signaling, we constructed ACS2-YFP and ACS6-YFP expressing vector driven by the constitutive CaMV 35S promoter, and generated transgenic lines overexpressing ACS2/ACS6 tagged with YFP (ACS2/ACS6-YFP). Then the transgenic lines were used to detect the response to TOR inhibitor AZD8055 in hypocotyl growth. As shown in Figure 6A–D, the transgenic lines overexpressing ACS2 or ACS6 displayed obviously constitutive triple response with shorter hypocotyl compared with WT etiolated seedlings, while exhibited more sensitive to AZD8055 in hypocotyl growth inhibition (Figure 6A–D). Specifically, the relative hypocotyl length of these transgenic lines ACS2-YFP-3, ACS2-YFP-9, ACS6-YFP-1 and ACS6-YFP-2 dropped to 38%, 29%, 37% and 33%, respectively, which were obvious lower than that of WT (about 45%) (Figure 6A–D). Previous reports suggest the ACS2 and ACS6 are short half-life protein and their stability is affected by post transcriptional regulation of phosphorylation [57,65]. Generally, ACS2 and ACS6 are degraded under favorable conditions, while accumulated in response to various environmental stimuli [57,58]. To further investigate whether TOR affects ACS2/ACS6 protein stability, we detected the protein level via western blotting in transgenic lines under TOR inhibition with AZD8055. As shown in Figure 6E, ACS2 protein was accumulated with increasing the AZD8055 dose (Figure 6E). Similar results are also observed in ACS6-YFP overexpressing lines (Figure 6F). These results indicate that TOR is involved in the regulation of ACS2/ACS6 stability.

## 3. Discussion

Target of rapamycin (TOR) plays a central role in integrating nutrient, energy, light and hormone signaling to regulate growth and metabolism by controlling key regulatory proteins for transcription and translation in plants [1,5,11,31,32]. However, our understanding of the roles of TOR signaling in crosstalk with plant hormone signaling is limited. In present study, through gene expression analysis, we found that TOR inhibition upregulated abundant senescence associated genes expression (Table S1, Figure 1A). Previous studies have reported that TOR functions in the regulation of senescence in plants [28,33,59,66]. Overexpression of TOR plants can dramatically accelerate senescence in leaves and siliques and suppression of TOR activity by rapamycin-FKBP12 leads to delayed senescence on culture plates [28]. However, TOR was reported to repress senescence mediated by TAP46, a downstream component of the TOR signaling, and ethanol inducible tor RNAi and *lst8* mutants also exhibited early senescence phenotype in *Arabidopsis* [59,66]. The reason for these inconsistent observations is likely that rapamycin-FKBP12 only partially reduces TOR activity. Nevertheless, AZD8055, one of the strongest and most selective inhibitors of TOR, can lead to remarkably senescence phenotype in plants [21,22,33]. Accordingly, senescence associated genes such as the marker genes (*SAG12*, *SAG13* and *ORE1*), companying with ethylene related genes including ethylene biosynthesis and response genes, showed consistently up-regulated expression (Table S1, Figure 1B). These results imply that ethylene may contribute to the senescence under TOR inhibition and TOR is involved in the regulation of ethylene signaling.

Seedling triple response, especially hypocotyl growth inhibition, are usually been used to evaluate ethylene response [64]. In the studies of TOR signaling, it has been observed that TOR regulates hypocotyl elongation and TOR inactivity by inhibitors or knockdown of expression, lead to dramatically reduction in hypocotyl length [28,35]. Previously, Glucose-TOR signaling promotes plant growth and hypocotyl elongation in darkness by activating BR pathway involved in BR-signaling transcription factor BZR1 stabilization [35]. The sugar promotion of hypocotyl elongation is blocked in the *tor-es* (estradiol-inducible TOR RNAi) seedlings or by TOR inhibitors treatment. On the other hand, crosstalk between glucose and ethylene signal also have been reported and glucose promotes degradation of EIN3, the key transcriptional factor in ethylene signaling [67]. These studies provide a hint that underlying link exists between TOR and ethylene signaling in regulation of hypocotyl growth. The link was supported by our results that the ethylene insensitive mutants exhibited hyposensitivity to AZD8055 in hypocotyl growth (Figure 2A,B), indicating that hypocotyl growth inhibition caused by TOR suppression is partly dependent on ethylene signaling. Previous works demonstrated that there exist distinct signal transduction pathways of ethylene especially in response to nutrient deficiencies such as Fe-deficiency [41,42,43,44,45]. In addition, it was reported that ethylene signals can be transduced to transcriptional events in both EIN2-dependent and -independent manners [68]. These studies indicate a possibility that TOR regulates hypocotyl growth via a EIN2-independent pathway. However, in terms of hypocotyl growth, the ethylene insensitive mutants exhibited similar response to TOR inhibitor AZD8055, suggesting that these mutants have identical signaling transduction to some extent. Additionally, the hyposensitive phenotype of *ein2-5* or *ein3 eil1* was inverted through overexpression of EIN2 or EIN3, respectively (Figure 3A–D), and blocking ethylene signals can alleviate hypocotyl growth inhibition under TOR suppression (Figure 4A–D). These results indicate that a classical ethylene is required for hypocotyl growth inhibition under TOR suppression and TOR participates in negative regulation of ethylene signals.

Considering that ACSs were the target of AVG, we speculate TOR directly modulated ACS activity in protein levels. In the present study, we demonstrated that a downstream effector of TOR, TAP46 physically interacted with ACS2/ACS6 (Figure 5C,D), implying the TAP46-ACS2/ACS6 interaction mediates the TOR signaling in regulation of ethylene. It was reported that TAP46, as a regulatory subunit of protein phosphatase 2A, can interacts with phosphatases PP4 and PP6, and modulates PP2A activities in plants [13,69]. On the other hand, RCN1-containing PP2A (RCN1 as the regulatory subunit) also was reported to negatively regulate ACS6 stability by dephosphorylating a C-terminal ACS6 phosphopeptide [65]. It is known that ACS is unstable in vivo, and phosphorylation of ACS increases its stability and activity while dephosphorylation leads to destabilization and degradation [57,65]. Therefore, our results of TAP46-ACS2/ACS6 interaction indicate a possibility that TAP46’s role in regulation of ACS2/ACS6 might involve PP2A phosphatase activities as well. The possibility also was enhanced by the results of that the accumulation of ACS2/ACS6 with the TOR inhibition (Figure 6E,F), though we could not define whether TOR inhibition changes the phosphorylated status of ACS2/ACS6 mediated through TAP46. Anyhow, these results will help us understand the underlying molecular mechanism linking TOR and ethylene signaling.

Collectively, we established the regulatory relationship between TOR ethylene signaling in *Arabidopsis*, in which TOR negatively modulates ethylene signals and the underlying molecular mechanism is likely involved in ethylene biosynthesis by regulating ACSs in transcription and protein levels. Our findings expand our understanding of TOR signaling in cross-talking with plant hormone signaling.

## 4. Materials and Methods

### 4.1. Arabidopsis Materials and Growth Conditions

All *Arabidopsis* thaliana plants used in this study were of the Columbia ecotype. The *Arabidopsis* materials included mutants *etr1-1* [70], *ein2-5* [51], *ein3 eil1* [71] and transgenic plants EIN3-GFP/dm (EIN3-GFP/*ein3 eil1*) [72]. In addition, BP12 (BP12-2 line), one of rapamycin hypersensitive transgenic lines created by Ren [28] through expressing yeast FKBP12 in *Arabidopsis*, also was used in this study. Seeds were surface-sterilized and then placed on half strength Murashige and Skoog (1/2 MS) solid medium containing 1% sucrose and 0.8% agar.

### 4.2. Plasmids Construction and Arabidopsis Transformation

To create EIN2-OE/*ein2-5*, ACS2/ACS6-YFP transgenic *Arabidopsis*, the coding sequence of EIN2, or ACS2/ACS6 fused YFP was cloned first on the entry vector p35S-8GWN, and then was cloned on plant binary vector KANA303 according to the experimental protocols described in previous reports [29]. The resulting destination vectors were transferred into Agrobacterium strain GV3101 for *Arabidopsis* transformation. The floral dipping method was employed for generating transgenic *Arabidopsis* [73], and T3 generation transgenic plants were used for subsequent assays. Primer sequences used in this part are listed in Supplementary Table S2a.

### 4.3. Inhibitor Treatments and Hypocotyl Growth inhibitionAssays

For the hypocotyl growth inhibition assays, seeds were incubated on 1/2 MS medium supplied with various inhibitor or chemicals such as 1μM AZD8055 (AZD), 10 μM AgNO3 (Ag+), 5 μM aminoethoxyvinylglycine (AVG) or their combination in the dark conditions for 5 days. Then, 0.1% dimethyl sulfoxide (DMSO) was taken as solvent control in the assays of AZD or rapamycin treatment. For the treatment of *ScFKBP12*-expressing plants, 1 μM rapamycin (RAP) was used. For hypocotyl length measurement, about 40 etiolated seedlings after 5 days of growth were photographed and the ImageJ bundled with 64-bit Java 1.8.0_112 (available online: http://imagej.nih.gov/ij/) was used to measure hypocotyl length according to the images of seedlings.

### 4.4. RNA Isolation and Gene Expression Analysis

Ten day old seedlings were transferred to 1/2 MS liquid medium with 2 μM AZD8055 or 0.1% DMSO (solvent control) for incubation as indicated time. After indicated time incubation, seedlings were collected for RNA extraction. Total RNA was isolated using an RNAprep Pure Plant Kit (TianGen, DP432, Beijing, China) and first-strand cDNA was synthesized with PrimeScript RT Reagent Kit with gDNA Eraser (Perfect Real Time) (TAKARA, RR047A, Otsu, Japan) following the manufacturer’s instructions. Real-time PCR was performed on the LightCycler 480 II System (Roche, Indianapolis, IN, USA) using the TB Green™ Premix Ex Taq™ (Takara, RR420A, Otsu, Japan). *ACTIN2* was used as internal control and primers used for qRT-PCR are listed in Supplementary Table S2b.

### 4.5. Yeast Two-Hybrid Assay

As for the Yeast two-hybrid assay, the full coding sequence of RAPATOR1, S6K1, S6K2 and TAP46 were cloned into pGBKT7 vector, and ACS2, ACS4 and ACS7 (Representing three Type of ACS, respectively) were cloned into pGADT7 vector, by seamless cloning using an In-Fusion HD Cloning Kit (Clontech, Mountain view, CA, USA) following the user’s manual. Yeast two-hybrid assay was performed following the manual’s instructions for the Yeastmaker Yeast Transformation System (Clontech, CA, USA). Different plasmids of pGBKT7 and pGADT7 combinations were co-transformed into the Y2H Gold strain via the PEG/LiAc transformation procedure. Yeast cells co-expressing the constructs were grown on basic media (SD-W-L), and then selected selective media (SD-W-L-H-A) with 125 μg/L Aureobasidin A and 40 mg/L X-α-gal. Primers used for construction of vector are listed in Supplementary Table S2c.

### 4.6. BiFC Assays

The vectors pXY104 and pXY106 that carry fragments encoding the carboxy (C)-terminal and amino (N)-terminal of YFP (cYFP and nYFP), respectively, were used to produce constructs for the BiFC assays [74,75]. The cDNA fragments encoding TAP46 fused with a HA tag was cloned into pXY106 to created construct pXY106-nYFP-TAP46-HA. The fragments encoding ACS2 and ACS6 with a MYC tag in front of ACS2/ACS6 were fused to the fragment encoding the C-terminus of YFP to generate the constructs pXY104-MYC-ACS2/ACS6-cYFP. All vectors were transformed into Agrobacterium strain GV3101. Agrobacterium cultures were grown 6 h in LB medium, then washed with infiltration medium (10 mM MgCl2, 10 mM MES, pH 5.8, 200 mM acetosyringone) and resuspended to an OD600 of 0.1. *Agrobacterium* strain GV3101 cultures harboring the nYFP and cYFP constructs were mixed with a volume ratio of 1:1, and the *Agrobacterium* cultures were introduced into lower surface of *N. benthamiana* leaves through infiltration. Then the transformed tobacco plants were incubated at 25 °C in the dark for 24 h following in the normal light cycles for 24 h, and then reconstituted fluorescence signals were detected by confocal microscopy (Olympus, Tokyo, Japan). Primers used in this part are listed in Supplementary Table S2d.

### 4.7. Co-IP Assays

Tobacco leaves were infiltrated with a mixture of *Agrobacterium* culture harboring the constructs expressing nYFP-TAP46-HA with MYC-ACS2-cYFP, MYC-ACS6-cYFP and MYC-YFP, respectively, at a volume ratio of 1:1. After 48 h of incubation, samples from tobacco leaves were collected and ground in liquid nitrogen, and 1 mL powder was homogenized in same volumes of RIPA buffer (Beyotime, Shanghai, China) containing 50 mM Tris (pH 7.4), 150 mM NaCl, 1% NP-40, 0.25% sodium deoxycholate, 1% (*v*/*v*) protease inhibitor cocktail and 1 mM phenylmethylsulphonyl fluoride (PMSF). The samples were incubated on ice for 30 min and then centrifuged at 12,000× *g* for 10 min at 4 °C twice. After centrifugation, protein supernatant was incubated with 20 μL of Anti-c-Myc Magnetic Beads (Pierce, Rockford, IL, USA) by tumbling end-over-end for 4 h at 4 °C. The immunoprecipitate was washed three times with 1 × TBST by inverting the tube several times, and then collect beads with a magnetic stand and discard the supernatant. The beads were resuspended with 50 μL 5 × SDS-PAGE loading buffer and heated in boiling water for 5 min. The eluted immunoprecipitates were immunoblotted with anti-HA and anti-MYC antibodies (EarthOx, San Francisco, CA, USA).

### 4.8. Western Blotting Analysis

Proteins were resolved by SDS-PAGE and electroblotted onto a PVDF membrane and probed with the indicated primary antibodies and then with secondary HRP-conjugated goat anti-mouse IgG (EarthOx, San Francisco, CA, USA). The signals were detected by a chemiluminescence reaction using the BeyoECL Moon Kit (Beyotime, Shanghai, China). Monoclonal anti-HA, anti-MYC and anti-GFP was used at dilution of 1:2000.

### 4.9. Data Processing

All presented results in this study were obtained from three independent experiments. Data were indicated as means ± standard deviations (SDs) of 40 etiolated seedlings (*n* ≥ 40) with each measurement performed in triplicate. The data were evaluated by a one-way analysis of variance (ANOVA), followed by Student tests at a significance level of *p* < 0.05 (*).

## Supplementary Materials

Supplementary Materials can be found at https://www.mdpi.com/1422-0067/21/8/2680/s1. Table S1: DEGs involved in senescence and ethylene signaling under TOR inhibition by AZD8055, Table S2: Primers used in this study.
