# Supplementary material for: Target of Rapamycin (TOR) Negatively Regulates Ethylene Signals in Arabidopsis
Source: Int J Mol Sci. 2020 Apr 12;21(8):2680. doi: 10.3390/ijms21082680 (PMC7215648; doi:10.3390/ijms21082680)
Supplement: Supplementary file 1 [file ijms-21-02680-s001.pdf]

Table S1 DEGs involved in senescence and ethylene signaling under TOR inhibition by AZD8055.

Name	Gene ID.	log2FC	Symbols
SAG12	AT5G45890	5.04	SENESCENCE-ASSOCIATED GENE 12
SAG13	AT2G29350	3.17	SENESCENCE-ASSOCIATED GENE 13
SAG14	AT5G20230	2.66	SENESCENCE ASSOCIATED GENE 14
SAG101	AT5G14930	1.37	SENESCENCE-ASSOCIATED GENE 101
SAG201	AT2G45210	2.00	SENESCENCE-ASSOCIATED GENE 201
SAG24	AT1G66580	1.05	SENESCENCE ASSOCIATED GENE 24
SAG15	AT5G51070	2.37	SENESCENCE ASSOCIATED GENE 15
SAG113	AT5G59220	1.62	SENESCENCE ASSOCIATED GENE 113
SAG2	AT5G60360	1.23	SENESCENCE ASSOCIATED GENE2
SAG20	AT3G10985	1.80	SENESCENCE ASSOCIATED GENE 20
SAG21	AT4G02380	1.94	SENESCENCE-ASSOCIATED GENE 21
SRG1	AT1G17020	3.01	SENESCENCE-RELATED GENE 1
SRG2	AT3G60140	2.45	SENESCENCE-RELATED GENE 2
SRG3	AT3G02040	1.07	SENESCENCE-RELATED GENE 3
SEN1	AT4G35770	3.02	SENESCENCE 1
SEN4	AT4G30270	3.37	SENESCENCE 4
SIRK	AT2G19190	3.87	SENESCENCE-INDUCED RECEPTOR-LIKE KINASE
MKK9	AT1G73500	1.69	MAP KINASE KINASE 9
MPK3	AT3G45640	2.65	MITOGEN-ACTIVATED PROTEIN KINASE 3
ACS6	AT4G11280	2.04	ACC SYNTHASE 6
ACS7	AT4G26200	3.47	ACC SYNTHASE 7
ACO4	AT1G05010	2.14	ETHYLENE FORMING ENZYME
ETR2	AT3G23150	1.26	ETHYLENE RESPONSE 2
CTR1	AT5G03730	1.17	CONSTITUTIVE TRIPLE RESPONSE 1
EBF2	AT5G25350	1.27	EIN3-BINDING F BOX PROTEIN 2
EDF1	AT1G25560	2.03	ETHYLENE RESPONSE DNA BINDING FACTOR 1
EDF2	AT1G68840	2.67	ETHYLENE RESPONSE DNA BINDING FACTOR 2
EDF4	AT1G13260	1.48	ETHYLENE RESPONSE DNA BINDING FACTOR 4
ERF1A	AT4G17500	2.77	ETHYLENE RESPONSIVE ELEMENT BINDING FACTOR 1
ERF1B	AT3G23240	3.84	ETHYLENE RESPONSE FACTOR 1
ERF2	AT5G47220	2.00	ETHYLENE RESPONSE FACTOR 2
ERF5	AT5G47230	1.85	ETHYLENE RESPONSIVE ELEMENT BINDING FACTOR 5
ERF6	AT4G17490	3.07	ETHYLENE RESPONSIVE ELEMENT BINDING FACTOR 6
ERF11	AT1G28370	2.60	ERF DOMAIN PROTEIN 11
ERF59	AT1G06160	1.79	ETHYLENE RESPONSIVE FACTOR 59
ERF98	AT3G23230	4.38	ETHYLENE RESPONSE FACTOR 98
ERF104	AT5G61600	2.58	ETHYLENE RESPONSE FACTOR 104
ERF105	AT5G51190	2.33	ETHYLENE RESPONSE FACTOR 105
ERF022	AT1G33760	5.41	ETHYLENE RESPONSE FACTOR 022
NAP	AT1G69490	3.19	ARABIDOPSIS NAC DOMAIN CONTAINING PROTEIN 29
ORE1	AT5G39610	2.53	ARABIDOPSIS NAC DOMAIN CONTAINING PROTEIN 92
NAC046	AT3G04060	2.86	NAC DOMAIN CONTAINING PROTEIN 46

Note: Original data seeing the reference (Dong et al., 2015. DEGs, Differentially expressed genes.

Table S2a Primers used for construction of *Arabidopsis* overexpression vector.

Assay	Constructs	Template genes	Primers	Primer sequences (5'-3')
Create transgenic *Arabidopsis*	EIN2-p8GWN/KANA303	EIN2	EIN2-F	GCGGCCGCATGGAAGCTGAAATTGTG
			EIN2-R	CCTGCAGGACCCAATGATCCGTACGCA
	ACS2-YFP-p8GWN/KANA303	ACS2-YFP	ACS2-YFP-F	GCGGCCGCATGGGTCTTCCGGGAAAAAATAAAG
			ACS2-YFP-M-R	CTCGCCCTTGCTCACCATTGCTCGGAGAAGAGGTGA
			ACS2-YFP-M-F	TCACCTCTTCTCCGAGCAATGGTGAGCAAGGGCGAG
			ACS2-YFP-R	CCTGCAGGCTTGTACAGCTCGTCCATG
	ACS6-YFP-p8GWN/KANA303	ACS6-YFP	ACS6-YFP-F	GCGGCCGCATGGTGGCTTTTGCAACAG
			ACS6-YFP-M-R	CTCGCCCTTGCTCACCATAGTCTGTGCACGGACTAG
			ACS6-YFP-M-F	CTAGTCCGTGCACAGACTATGGTGAGCAAGGGCGAG
			ACS6-YFP-R	CCTGCAGGCTTGTACAGCTCGTCCATG

Table S2b Primers used for qRT-PCR.

Gene	Accession no.	Forward (5'-3')	Reverse (5'-3')
SAG12	AT5G45890	GAGGTGGTTTTGATTTCC	CAGTTGGAGGCAATGTGT
SAG13	AT2G29350	AGATGGAGTCTTGGAGGCA	TTGTTGACGAGGATGTTGAG
ORE1	AT5G39610	TGAAAATCTTCCCCAAAC	ACAGAGAACCAAAACCGT
ACS2	AT1G01480	GCCAACATGGACGATGATAC	TCCCTGGAGACGAGAGACC
ACS6	AT4G11280	GTTTCGGTCTTGTTTCGTC	GTGGTTATCTCAGCGTGCC
ACO2	AT1G62380	ACAAAGGACCATTACAAGACA	ATTGAGGGAGGTGACGAAC
ACO4	AT1G05010	AAGCACCTTCCCGTCTCT	TACTTCCCATTGGTTATCAC
ERF1	AT3G23240	AGCAGTCCACGCAACAAAC	CCGAGCCAAACCCTAATAC
EBF2	AT5G25350	TGCTCTGGTGTTGGGAATG	CATCTGGAGTTTCACTTTCG
EIN2	AT5G03280	AAGCCTTACAATCCCGTGG	AACCGACCATAAGACGCA
ACTIN2	AT3G18780	CTGTGCCAATCTACGAGGGT	GAGCTGGTCTTTGAGGTTTCC

Table S2b Primers used for construction of Y2H assays vector.

Assay	Constructs	Template genes	Primers	Primer sequences (5'-3')
Y2H	pGBKT7-RAPTOR1	RAPTOR1B	RAPTOR1-BD-F	ATGGAGGCCGAATTCATGGCATTAGGAGACTTAATG
			RAPTOR1-BD-R	CAGGTCGACGGATCCCTATCTTGCTTGCGAGTTGTCG
	pGBKT7-S6K1	S6K1	S6K1-BD-F	ATGGAGGCCGAATTCATGGTTTCCTCTCAGCGTCC
			S6K1-BD-R	CAGGTCGACGGATCCCTACAAAGTAGTTGTGG
	pGBKT7-S6K2	S6K2	S6K2-BD-F	ATGGAGGCCGAATTCATGGTTTCTTCTCAGTGTTC
			S6K2-BD-R	CAGGTCGACGGATCCCTACAAGTTGGATGTGGTCC
	pGBKT7-TAP46	TAP46	TAP46-BD-F	ATGGAGGCCGAATTCATGGGTGGTTTGGCTATGGA
			TAP46-BD-R	CAGGTCGACGGATCCTCAGCCACAAGGTGTGAGT
	pGADT7-ACS2	ACS2	ACS2-AD-F	GAGGCCAGTGAATTCATGGGTCTTCCGGGAAAAAATAAAG
			ACS2-AD-R	GAGCTCGATGGATCCTCATGCTCGGAGAAGAGGTG
	pGADT7-ACS4	ACS4	ACS4-AD-F	GAGGCCAGTGAATTCATGGTTCAATTGTCAAGAAAAG
			ACS4-AD-R	GAGCTCGATGGATCCCTATCGTTCCTCAGCCTCAC
	pGADT7-ACS7	ACS7	ACS7-AD-F	GAGGCCAGTGAATTCATGGGTCTTCCTCTAATGA
			ACS7-AD-R	GAGCTCGATGGATCCTCAAAACCTCCTTCGTCGGTCC

Table S2c Primers used for construction of BiFC assays vector.

Assay	Constructs	Template genes	Primers	Primer sequences (5'-3')
BiFC and Co-IP	pXY106-nYFP-TAP46-HA	TAP46-HA	TAP46-HA-F	GACGCCGGCGGATCCATGGGTGGTTTGGCTATG
			TAP46-HA-M-R	AGCGTAATCTGGAACATCGTATGGGTAGCCACAAGGTGTGAGTTTC
			TAP46-HA-R	CAGGTCGACTCTAGATCAAGCGTAATCTGGAACATCG
	pXY104-MYC-ACS2-cYFP	MYC-ACS2	MYC-ACS2-F	GGTACCCGGGGATCCATGGAGCAGAAACTCATC
			MYC-ACS2-M-F	ATGGAGCAGAAACTCATCTCTGAAGAGGATCTGATGGGTCTTCCGGGAA
			MYC-ACS2-R	GCCGTCGACTCTAGATGCTCGGAGAAGAGGTGA
	pXY104-MYC-ACS6-cYFP	MYC-ACS6	MYC-ACS6-F	GGTACCCGGGGATCCATGGAGCAGAAACTCATC
			MYC-ACS6-M-F	ATGGAGCAGAAACTCATCTCTGAAGAGGATCTGATGGTGGCTTTTGCAACAG
			MYC-ACS6-R	GCCGTCGACTCTAGAAGTCTGTGCACGGACTAG
	pXY104-MYC- YFP	MYC-nYFP	MYC- nYFP-F	GGTACCCGGGGATCCATGGAGCAGAAACTCATC
			MYC- nYFP-M-F	ATGGAGCAGAAACTCATCTCTGAAGAGGATCTGATGGTGAGCAAGGGCGA
			MYC- nYFP-M-R	GCCGTCGACTCTAGAGTCCTCGATGTTGTGGC
